# Study on the associations between liver damage and antituberculosis drug rifampicin and relative metabolic enzyme gene polymorphisms

**DOI:** 10.1080/21655979.2021.2003930

**Published:** 2021-12-07

**Authors:** Qiang Su, Qiao Liu, Juan Liu, Lingyun Fu, Tao Liu, Jing Liang, Hong Peng, Xue Pan

**Affiliations:** a Department of Pharmacy, Nanchong Central Hospital, the Second Clinical Medical College, North Sichuan Medical College, Nanchong, P.R. China; b Nanchong Key Laboratory of Individualized Drug Therapy, Nanchong, P.R. China; c School of Pharmacy, North Sichuan Medical College, Nanchong, P.R. China; d Department of Pediatrics, Nanchong Central Hospital, the Second Clinical Medical College, North Sichuan Medical College, Nanchong, P.R. China; e Department of Health Management Center, Nanchong Central Hospital, the Second Clinical Medical College, North Sichuan Medical College, Nanchong, P.R. China; f Department of Cardiology, Nanchong Central Hospital, the Second Clinical Medical College, North Sichuan Medical College, Nanchong, P.R. China; g Department of Anorectal Surgery, Nanchong Central Hospital, the Second Clinical Medical College, North Sichuan Medical College, Nanchong, P.R. China; h Scientific Research Department, The First Affiliated Hospital of Chongqing Medical University, Chongqing, P.R. China

**Keywords:** Tuberculosis, liver damage, rifampicin, gene polymorphisms

## Abstract

The occurrence of antituberculosis drug-induced liver injury affects the effectiveness of antituberculosis treatments. Understanding the mechanism and risk factors of such liver injury may improve the outcomes of those patients who received antituberculosis treatments. In this study, 2,255 pulmonary tuberculosis patients were included. Their medical records were reviewed, questionnaire surveys, liver function tests at the end of February (including patients with uncomfortable symptoms during the intensive treatment period), and blood samples were saved. Afterward, cases of liver damage were determined using Chinese liver damage criteria. The genotype of all participants was determined using the PCR-LDR method. Finally, the association between genetic polymorphism and ATB-DILI susceptibility was assessed using the univariate Logistic regression models. Among the 2,255 tuberculosis patients who received rifampicin, 612 (27.1%) had antituberculosis drug-induced liver injury. We observed higher proportions of older age, male, and lower levels of AST, ALT, and TBil among patients with liver injury. Results of univariate of logistic regression models showed that patients with CYP2C19 were more likely to have liver injury compared with no such genotypes patients (all *P* < 0.05). Patients with tuberculosis with older age and genetic polymorphism of CYP3A4, CYP2C9, and CYP2C19 who received long-term rifampicin treatment were more likely to have antituberculosis drug-induced liver injury. It is important for healthcare providers to carefully evaluate and monitor rifampicin use for these patients.

## Introduction

Tuberculosis (TB) is a chronic infectious disease mainly transmitted by the respiratory tract and has severely harmed human health for thousands of years [1,2]. To date, it is still a major global public health and social problem [3–8]. The core treatment of the directly observed treatment, short-course (DOTS) strategy is standard short-term chemotherapy based on the reasonable combination of sterilization, bacteriostasis, and prevention of drug resistance [9,10]. Three first-line drugs, including isoniazid (INH, H), rifampicin (RIF, R) and pyrazinamide (PZA, Z), are recommended by the WHO as standard short-term chemotherapy regimens. However, these medications can cause adverse drug reactions (ADRs) of different degrees and frequencies while killing tuberculosis [11]. The main adverse reactions are gastrointestinal reaction, liver damage, allergic reaction, auditory nerve damage, optic nerve damage, and kidney damage. The most common adverse reaction is the gastrointestinal reaction. The more severe and frequent adverse reaction is liver damage. The resulting deaths have also been reported. The incidence of liver damage caused by antituberculosis drugs varies from 2.5% to 34.9% [12]. The reasons for the different incidences of liver damage caused by antituberculosis drugs in different countries and regions may be associated with different treatment regimens, different criteria for judging whether the liver damage caused by antituberculosis drugs occurs or not and its severity, as well as demographic differences. According to many studies, the occurrence of liver damage caused by antituberculosis drugs not only affects the medication compliance of patients but also boosts the risk of multidrug resistance due to irregular medication [13]. For example, the delay of treatment or aggravation of the disease owing to drug withdrawal may lead to the failure of short-term chemotherapy and even death of patients, resulting in a negative impact on the implementation of tuberculosis prevention and control plan in China [14]. Therefore, the mechanism and predisposing factors of liver damage caused by antituberculosis drugs must be revealed to reduce or avoid the occurrence of liver damage and complete the antituberculosis treatment smoothly.

The mechanism of antituberculosis drug-induced liver injury (ATB-DILI) has not been fully elucidated. At present, some studies suggest that INH, RIF, and PZA are the main antituberculosis drugs with potential hepatotoxicity [15]. These enzymes are involved in the production and metabolic excretion of toxic intermediates. Therefore, the activities of metabolic enzymes such as NAT2, CYP, and GST would play a crucial role in the occurrence of ATB-DILI. Besides, the association between genetic polymorphisms of the genes encoding these drug metabolizing enzymes (DMEs) and ATB-DILI has recently become a hot topic in pharmacogenomics in the post-genomic era. Actually, previous studies indicated several variants, such as CYP3A4, CYP2C9, and CYP2C19, are associated with drug metabolism. One explanation is that these variants confer reduced human cytochrome P450 function. However, there is lacking relative evidence of such variants in antituberculosis drugs.

We hypothesize tuberculosis patients with genotyping CYP3A4 enzyme, CYP2C9 enzyme, and CYP2C19 enzyme are more likely to have ATB-DILI. Therefore, this study aims to assess the associations between ATB-DILI susceptibility and gene polymorphism from the perspective of genetics variants and enzyme variants.

## Methods

### Data source and study population

In this study, we included 2255 patients with pulmonary tuberculosis who received antituberculosis treatments between 1 January 2015 and 31 December 2019 in the Nanchong Central hospital, Sichuan province, China. A total of 612 patients were included in the ATB-DILI case group, and 1643 patients with normal liver function were included in the control group. A total of 144 patients with abnormal liver function were excluded according to the criteria of ATB-DILI. This study was approved by the medical Ethics Committee of our hospital, and all patients signed informed consent.

Inclusion criteria: (1) the diagnosis of pulmonary tuberculosis was based on the standard of ‘guidelines for diagnosis and treatment of pulmonary tuberculosis’ formulated by the tuberculosis branch of Chinese Medical Association in 2001 [16], (2) patients with newly diagnosed and retreated pulmonary tuberculosis were all included, (3) liver function was completely normal before antituberculosis treatment. The liver function was evaluated based on the three liver tests during the study time. All participants needed to sign the free treatment agreement of China tuberculosis control program and accepted for 6 months or more therapy. All patients voluntarily participated in the survey, and have signed informed consent forms, clinical data of all patients were completely abstracted by trained nurses and physicians. All patients received only first-line antituberculosis treatment, including INH, RIF, and PZA.

Exclusion criteria: (1) those did not meet the national conditions for free treatment; (2) or those had mental or disability diseases and could not accept the survey; (3) or those were unwilling to participate in the study for some reasons; (4) or there are tumors, diabetes, hepatitis, and other diseases that may cause liver dysfunction; (5) or those had an abnormal liver function before antituberculosis treatment; (6) or patients survived less than 6 months; (7) or those had a history of habitual drinking; (8) or those patients are allergic to INH, RFP, and other first-line antituberculosis drugs were excluded.

### Therapeutic schedule

All patients were prescribed Isoniazid (INH, 600 mg), rifampin (RMP, 600 mg, or 450 mg if the body weight was less than 50 kg), pyrazinamide (PZA, 2,000 mg), and ethambutol (EMB) (1,250 mg) every other day in the first 2 months [17]. After 2 months, INH and RMP were continued for a further 4–6 months. Retreated patients received streptomycin (SM) (750 mg) every other day in the first 2 months and continued receiving EMB therapy for another 6 months. When patients developed a suspected adverse drug reaction (ADR) (ATB-DILI, gastrointestinal reaction, allergic reaction, nervous system disorders, or arthralgia), their treatment was adjusted according to the severity of the symptoms. This study has been approved by the Institutional Review Board of Nanchong Central hospital.

### Definition of ATB-Dili

In the primary analysis, ATB-DILI was defined as an aspartate transaminase (ALT), aspartate aminotransferase (AST), or bilirubin value more than two times the upper limit of normal value. The upper limit of normal use in the study was 40 U/L, 40 U/L, and 19 µmol/L for ALT, AST, and total bilirubin, respectively. In secondary analyses, alternative case definitions were considered. First, ATB-DILI was defined as values above three or above 5 times the upper limit of normal use. Second, it is defined as ATB-DILI if the patient’s ALT, AST, or bilirubin level corresponds to a grade 3 or 4 adverse event in the AIDS classification of adult and child adverse event severity table [18].

### DNA extraction from human peripheral blood

Blood samples from the above subjects were stored at −20°C or below and dissolved at room temperature. Whole-blood genomic DNA extraction kit (Tiangen Biochemical Technology Co., Ltd. DP319) was used to extract genomic DNA from 5 ml venous blood according to the instructions. The whole blood genomic DNA extraction process is described below. First, 5 ml of anticoagulant was added to the blood samples dissolved at room temperature, and an equal volume of cell lysate CL was added. Mix inversely 5 times, 8000 rpm/separate for 2 min. Ditch the supernatant. Then, 7.5 mL cell lysate CL was added and mixed upside-down for five times, followed by centrifugation at 8000 rpm for 2 min. Discard the supernatant. Placed the tube upside down on a clean blotting paper for 2 min to ensure that the sediment settles in the tube. The mixture of buffer FG and Proteinase K was prepared. Added another 2.5 ml of the mixture and stir until no agglomeration occurs. Place the mixture in a 65°C water bath for 10–30 min and invert it several times during this time. Then, 2.5 ml isopropyl alcohol was added and thoroughly mixed until filamentous or clumped genomic DNA was present. Centrifuge at 8000 rpm for 8 min and slowly discard the supernatant. The centrifuge tube is placed upside down on a clean sheet of blotting paper to ensure that it settles in the tube. 2.5 mL 70% ethanol was added, followed by vortex oscillation for 5 s and centrifugation at 8000 rpm for 3 min. Ditch the supernatant. Then place the tube upside down on clean blotting paper for 5 min to make sure the sediment stays in the tube. The DNA deposits are air-dried until all the liquid evaporates (at least 5 min). Then 500 μL buffer TB was added, and DNA was dissolved in water bath at 65°C for 1 h at low eddy speed for 5 s. DNA was quantified by ultraviolet spectrophotometer. The OD values were measured at 260 nm and 280 nm, and the concentration and purity of DNA were calculated.

### PCR-LDR method to determine the genotype

In this section, 50 μl of peripheral blood DNA extraction product was taken for genetic polymorphism detection. The presence or absence of CYP3A4 *5/*18, CYP2C9 *2/*13 CYP2C19 *2/*3 was selected from the NCBI dSNP database. Oligo 6.0 software was employed to design and synthesize primers (Table 1). According to the molecular weight of the primers and probes, it was diluted with TE to 50 pmol/µl as the mother solution. In the case of multiplex PCR and LDR, the mother solutions of the primers are mixed in equal proportions to form a mixture solution that can be directly reacted. Afterward, 3% agarose gel electrophoresis was used to detect the effect of the PCR product and determine the amount of it as a template in the LDR reaction.

**Table 1.** Primer sequences

Primers	Forward	Reverse
CYP3A4 *5	TGCATTTTCTGCGTGACAGAAG	AGGAACGAAATGATGTGGTTAT
CYP3A4 *18	AAGTGATTTGGCTGGATTG	AGACCCTCTTCCACCTTCT
CYP2C9 *2	TCAGAGTTTCTGGGGAAGC	GAGGGTAGAGAGGATATCTGATA
CYP2C9 *13	TTGCTCGAGGACAAGTTC	TCTGATTTGGGGACCACAG
CYP2C19 *2	AGCTGCACTGTGACAAGCT	CATTCGTCTGTTTCCCATT
CYP2C19 *3	CATTTTCTGCTTGACAGAAGA	GGAACAAAATGATGTGGTTAT

## Other variables

Demographic and clinical characteristics at the baseline were manually abstracted for all participants from the medical records, and included age, sex, race, body mass index (BMI), smoking status, and drinking status.

## Statistical methods

Baseline patients and treatment characteristics were compared using Fisher’s exact test for categorical variables and *t*-test or rank-sum test for continuous variables across patients with and without ATB-DILI. Then, Haploview 4.2 software and Phase 2.1 software were adopted to construct haplotypes. Besides, the relationship between the genotype distribution of each polymorphic locus and the risk of ATB-DILI was analyzed using univariate and multivariate unconditional logistic regression with and 95% confidence intervals (CIs). All tests are two-sided tests, and the test level is taken as 0.05. Moreover, *P* ≤ 0.05 suggests that the difference is statistically significant.

## Results

### General situation of the research object

Overall, the results of this study supported the hypothesis of tuberculosis patients with several genotypes were more likely to have ATB-DILI. Based on our retrospective correctional study using electronic medical records in the Nanchong Central hospital, we found patients with tuberculosis with older age and genetic polymorphism of CYP3A4, CYP2C9, and CYP2C19 who received long-term rifampicin treatment were more likely to have antituberculosis drug-induced liver injury.

Among the study sample (Table 2), there were 612 (27.1%) patients with tuberculosis who had ATB-DILI after receiving antituberculosis treatments. Patients who had ATB-DILI tend to be older, and higher BMI (all *P* < 0.05) (Table 2).

**Table 2.** General comparison of ATB-Dili and control group

Features	Case	Control	*P* value
Case	612 (27.1)	1643 (72.9)	
Men	362 (59.2)	1049 (63.8)	0.04
Age	73.28 (12.92)	67.55 (12.51)	<0.001
Age groups			<0.001
<60	80 (13.1)	422 (25.7)	
60–	131 (21.4)	467 (28.4)	
70–	175 (28.6)	436 (26.5)	
80–	226 (36.9)	318 (19.4)	
BMI, mean (SD), kg/m^2^	24.57 (4.56)	24.84 (4.27)	0.494
Smoking in past/now	220 (36.1)	693 (42.4)	0.506
Drinking in past/now	64 (10.5)	252 (15.5)	0.473
AST (U/L)	1.24 (0.76)	1.43 (0.93)	<0.001
ALT (U/L)	1.09 (0.63)	1.09 (0.57)	0.026
TBil (U/L)	2.70 (0.98)	2.73 (1.00)	0.015

**Abbreviations**: ATB-DILI, anti-TB drug-induced liver injury; BMI, body mass index; SD, standard deviation; ALT, alanine aminotransferase; AST, aspartate aminotransferase.

### The PCR amplification products of the SNPs in CYP3A4, CYP2C9 and CYP2C19

**Figure 1.**
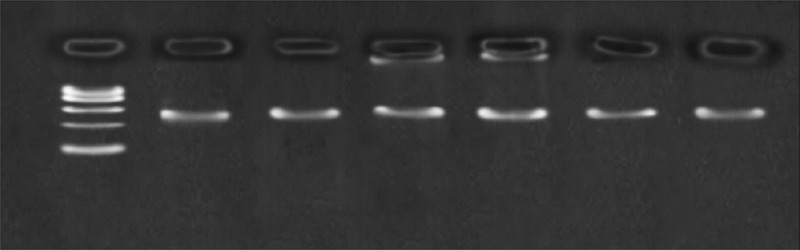
Detection of SNP PCR product in agarose gel electrophoresis

Through 3% agarose gel electrophoresis detection, the effect of the PCR product was observed, and the amount of it was determined as a template in the LDR reaction. Figure 1 illustrates the PCR electrophoresis diagram (the main observation in the experiment is whether the PCR is successful and whether each site is good cannot be observed by electrophoresis): Marker: 100, 200, 300, 400, 500, 6000.

**Figure 2.**
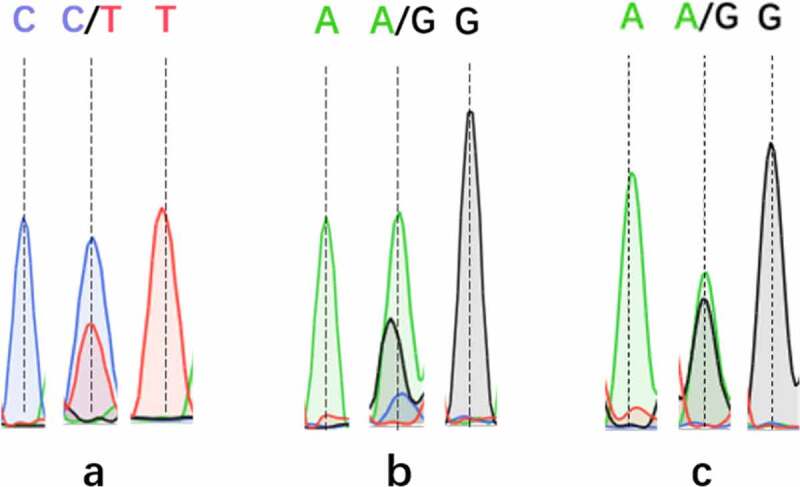
DNA sequencing peak chromatograms for the CYP3A4, CYP2C9, and CYP2C19 genotyping, dotted line indicates the mutation site: (a) CYP3A4 polymorphism, (b) CYP2C9 polymorphism, (c) CYP2C19 polymorphism

The SNP typing results of ABI 3130XL demonstrated that there were four genotypes for each SNPs of CYP3A4, CYP2C9, and CYP2C19: T, CT, CC, and C for CYP3A4, GG, GA, A and G for CYP2C9, GA, AA, G and GG for CYP2C19. DNA sequencing peak chromatograms for these genotyping results are presented in Figure 2(a,b and c).

### Hardy–Weinberg balance test

The Hardy–Weinberg equilibrium test was performed on the polymorphic loci of the RIF gene, as exhibited in Table 3. It can be observed that the genotype at CYP3A4 *5/*18 can satisfy the Hardy–Weinberg equilibrium (*P* > 0.05), the genotype at CYP2C9 and CYP2C19 does not reach the Hardy–Weinberg equilibrium (*P* < 0.01).

**Table 3.** Hardy–Weinberg test of the genotype distribution of polymorphic loci in the control group

Polymorphic site	Actual frequency	Theoretical frequency	*P* value
CYP3A4			
T	110.54 (65.75)	103.28 (64.43)	0.019
CT	137.51 (5.80)	138.15 (8.97)	0.106
CC	4.26 (1.19)	4.35 (1.26)	0.141
C	9.01 (4.22)	7.82 (3.06)	<0.001
CYP2C9			
GG	214.39 (83.14)	207.12 (72.23)	0.044
GA	74.67 (40.96)	80.35 (36.98)	0.002
A	4940 (9194)	28,545 (5218)	<0.001
G	226 (36.9)	318 (19.4)	0.374
CYP2C19			
GA	24.57 (4.56)	24.84 (4.27)	0.494
AA	220 (36.1)	693 (42.4)	<0.001
G	64 (10.5)	252 (15.5)	0.473
GG	2.70 (0.98)	2.73 (1.00)	0.015

### Genotype distribution of RIF polymorphic loci

Table 4 provides the distribution of RIF polymorphism sites in the ATB-DILI group and the control group. As revealed from the table, the proportion of wild type at the above three RIF polymorphic sites in the ATB-DILI group and control group is higher than that of heterozygous type, and heterozygous type is higher than that of mutant homozygous type. Only the different genotypes at the CYP2C9 locus are observed in the ATB-DILI group and control group. The distribution difference between the groups was statistically significant.

**Table 4.** Genotype distribution of RIF polymorphic loci

Clinical prognosis	Case	Control	*X*^2^ (95% CI)	*P* value
CYP3A4				
T	133 (21.7)	202 (12.3)	1.981 (1.555, 2.523)	0.144
CT	156 (25.6)	260 (15.8)	1.820 (1.453, 2.279)	0.245
CC	75 (12.3)	128 (7.8)	1.653 (1.223, 2.235)	0.04
C	139 (22.7)	303 (18.4)	1.300 (1.036, 1.630)	0.073
CYP2C9				
GG	142 (23.2)	321 (19.5)	1.244 (0.995, 1.556)	<0.001
GA	155 (25.3)	358 (21.8)	1.217 (0.980, 1.512)	<0.001
A	199 (32.5)	347 (21.1)	1.800 (1.464, 2.212)	<0.001
G	156 (25.6)	260 (15.8)	1.820 (1.453, 2.279)	<0.001
CYP2C19				
GA	64 (10.5)	94 (5.7)	1.925 (1.380, 2.683)	0.053
AA	99 (16.2)	167 (10.2)	1.706 (1.304, 2.230)	0.481
G	116 (19.0)	215 (13.1)	1.553 (1.212, 1.990)	0.653
GG	199 (32.5)	347 (21.1)	1.800 (1.464, 2.212)	0.287

**Abbreviations**: RIF, rifampicin; CI, confidence interval.

### Association between the genotype of RIF polymorphism and ATB-Dili

The RIF gene is mainly mutated at three sites: CYP2C9*2, CYP2C9*13, and CYP2C19*2. Univariate and multivariate unconditional Logistic regression was employed to analyze the association between different genotypes (additive models) of RIF polymorphism sites and ATB-DILI. The univariate analysis only indicated that CT genotype at CYP2C9*2 was significantly associated with ATB-DILI, with an OR value of 1.198 (1.079, 1.372). After adjusting for gender, age, and region, the results of multivariate analysis only found that the GA genotype at CYP2C9*2 locus was significantly correlated with ATB-DILI, with an OR value of 1.214 (1.096, 1.535). The results are presented in Table 5. For the three SNPs at RIF polymorphism sites, the relationship between the genetic model of these three SNPs and ATB-DILI in the dominant model and recessive model was analyzed using single factor and multivariate unconditional logistic regression. The results of single-factor and multivariate analysis suggested that CYP2C9*2 has an association with ATB-DILI in the dominant model, and the OR values are 1.198 (1.079, 1.372) and 1.214 (1.960, 1.535). Moreover, no association between genetic models at other sites and ATB-DILI was observed (All *P* > 0.05).

**Table 5.** The genotypes of RIF polymorphism loci, the association between different genetic models and ATB-Dili

Polymorphic site	OR (95% CI)
CYP3A4	
T	1.609 (1.082,2.391)
CT	1.454 (1.067,1.982)
CC	1.139 (0.902,1.438)
C	1.111 (0.883,1.399)
CYP2C9	
CT	1.198 (1.079,1.372)
GA	1.214 (1.096,1.535)
A	1.475 (0.952,2.284)
G	1.540 (1.094,2.168)
CYP2C19	
GA	1.394 (1.057,1.838)
AA	1.264 (0.978,1.633)
G	1.609 (1.082,2.391)
GG	1.454 (1.067,1.982)

**Abbreviations**: ATB-DILI, Anti-TB drug-induced liver injury; OR, odds ratio; RIF, rifampicin; CI, confidence interval.

## Discussion

Haplotype, as a relatively common data type, plays an essential role in the study of genetic epidemiology, including the association analyses for finding and locating disease-causing genes [19]. A single nucleotide polymorphisms (SNPs) site provides extremely limited information for complex diseases because of the interactions among multiple sites [20]. Many reports indicated that haplotypes contain more Linkage Disequilibrium (LD) information, which is conducive to finding and locating pathogenic sites in association analysis. Therefore, studies based on haplotypes are better than studies based on SNPs and finally get more solid evidence [21,22].

As revealed through the screening of related factors affecting ATB-DILI, the RIF gene is mainly mutated at three sites: CYP2C9*2, CYP2C9*13, and CYP2C19*2. CYP2C9 and CYP2C19 are located in a set of cytochrome P450 genes on chromosome 10q24. In addition, only the different genotypes at the CYP2C9 locus were observed in the ATB-DILI group and control group. The univariate analysis suggested that CT genotype at CYP2C9*2 was significantly associated with ATB-DILI. This association was still significant after controlling other covariates. For the three SNPs at RIF polymorphism sites, CYP2C9*2 has an association with ATB-DILI in the dominant model, and the OR values are 1.198 and 1.214, as demonstrated by the results of single-factor and multivariate analysis. However, the results of this association are still inconsistent. For example, *Tang* and the colleagues [23] compared 89 ATB-DILI patients and 356 controls in the Chinese community and found no significant association between ATB-DILI risk and genetic polymorphisms for CYP3A4, CYP2C9, and CYP2C19. In their study, they also did not find any of the haplotypes significantly associated with ATB-DILI development. Similarly, Pachkoria and colleagues [24] did not observe CYP2C9 and CYP2C19 gene polymorphisms could be risk factors of DILI. Besides a larger sample size, compared with previous studies, we speculated that CYP3A4, CYP2C9, and CYP2C19 were involved in RIF metabolism [25]. However, the gene polymorphism had little effect on the transcription of the gene encoding the enzyme. In addition, different antituberculosis drugs have different degrees of dependence on a single CYP in hepatocytotoxicity. Troglitazone is more dependent on CYP3A4, while tienilic acid, valproic acid, and diclofenac are more dependent on CYP2C9 [26]. Apart from CYP450, SNPs in the drug transporter and vitamin D pathways would also influence rifampicin pharmacokinetics and thus lead to an increased risk of DILI [27].

One potential explanation of the association between CYP3A4, CYP2C9, and CYP2C19 and ATB-DILI is related to drug metabolism. Oxidation, reduction, and hydrolysis are the main pathways of drug metabolism before excretion. The most imperative enzyme system for phase I metabolism is the CYP system, a microsomal isozyme superfamily, that catalyzes the oxidation of many drugs [28]. Nevertheless, many studies on ATB-DILI are based on the metabolic pathway of isoniazid, but the pathogenic mechanism of ATB-DILI remains unclear. In the liver, isoniazid is first metabolized to acetyl isoniazid through NAT2 and then hydrolyzed to acetyl isoniazid [29]. Acetyl hydrazine is oxidized to hepatotoxic intermediates mainly through CYP2E1, which is the main catalytic enzyme for the formation of hepatotoxin [30]. RIF is a macromolecular drug, which is mainly excreted via bile, and its own liver toxicity is relatively low. The main metabolic pathway of RIF is deacetylation to produce 25-O-desacetylrifampicin, which is then hydrolyzed to produce 3-formyl rifampicin. Most of them are secreted into bile through the bile duct and excreted into the small intestine, part of them enter enterohepatic circulation and are finally excreted with 25-desacetyl rifampicin. Studies have revealed that the metabolites of RIF have no significant hepatotoxicity [31]. The liver damage caused by RIF is primarily hepatic and cholestatic mixed hepatitis changes, leading to an increase in the serum bilirubin level and transaminase level. RIF interferes with the binding of bilirubin and glucuronic acid in liver cells and competes with bilirubin for excretion, increasing the unbound and bound bilirubin in the blood, causing cholestatic liver damage, and boosting the degree of dose-related damage [32]. Simultaneously, RIF could also induce various metabolic enzymes in the liver [33], aggravating the toxicity of combined drugs. When it is combined with INH, the risk of liver injury will increase [34]. Moreover, RIF can cause type IV hypersensitivity and liver damage. Studies have discovered that RIF, as an inducer of cytochrome P450 [35], could increase the toxic metabolites of INH and significantly aggravate the liver damage of INH [36]. These studies have verified that RIF-induced liver injury was closely related to CYP. Therefore, these studies may indirectly to support our study.

In addition, normal enzyme function is related to CYP2C9*1, but the two most common allelic variants, CYP2C9*2 and CYP2C9*3 may reduce the enzyme activity of 30% and 80%, respectively. In previous studies, up to 39% Chinese individuals had CYP2C9*2 variant. This may partially explain the reasons of high ATB-DILI incident rate in Chinese population.

This study has several strengths. First, the ATB-DILI cohort and the referent cohort were representative of a well-defined population who received antituberculosis treatments. Second, details about the ATB-DILI, treatments, baseline characteristics, and relative biomarkers were confirmed through abstraction of medical records, thus limiting recall bias. Third, compared with previous studies, our sample size was larger.

However, there are some deficiencies in this study. In this study, only the association between CYP and RIF in ATB-DILI patients was verified. The mechanism of CYP3A4, CYP2C9, and CYP2C19 mediated RIF ATB-DILI should be further investigated. Additionally, not all CYP enzymes were analyzed in this study. Thus, more studies are required to perfect the role of the CYP enzyme in RIF-induced ATB-DILI. Moreover, other first-line antituberculosis drugs should also be explored.

## Conclusion

There is an association between the genetic polymorphism site CYP2C9*2 and ATB-DILI, and the best association model is a dominant inheritance model. An in-depth discussion of the genetic nature of ATB-DILI could significantly and practically contribute to preventing the occurrence of liver damage in patients with tuberculosis in our region, reducing the damage to the liver by antituberculosis drugs, and formulating individualized chemotherapy regimens, so as to avoid treatment failure due to liver damage.

## Abbreviations

TB, Tuberculosis; INH, isoniazid; RIF, rifampicin; PZA, pyrazinamide; ADRs, adverse drug reactions; DMEs, drug metabolizing enzymes; EMB, ethambutol; SM, streptomycin; CI, Confidence Interval; OR, odds ratio; LD, Linkage Disequilibrium.
